# Boundary-Transition Characterization in High-Speed Underwater Bubble Imaging Under Broadband and Narrow-Band Illumination

**DOI:** 10.3390/s26175546

**Published:** 2026-08-31

**Authors:** Chen Lu, Songtao Fan, Yuang Yang, Jun He, Yang Cao

**Affiliations:** 1 School of Instrumentation Science and Opto-Electronics Engineering, Beijing Information Science & Technology University, Beijing 100192, China; luchen@semi.ac.cn (C.L.); yangyuang@semi.ac.cn (Y.Y.); 2 Optoelectronic System Laboratory, Institute of Semiconductors, Chinese Academy of Sciences, Beijing 100083, China; fstao@semi.ac.cn (S.F.); junhe@semi.ac.cn (J.H.)

**Keywords:** underwater bubble imaging, high-speed imaging, gas–liquid interface, narrow-band illumination, relative boundary width, boundary localization

## Abstract

**Highlights:**

**What are the main findings?**

- Within the sampled 5.0 m run, the blue-source condition had the lowest observed mean RBW of 17.46%, whereas the near-field mean RBW values were similar across source conditions.
- The image-reference benchmark did not support superior boundary localization, and RBW showed little monotonic association with normalized image-reference localization discrepancy in the holdout set.

**What are the implications of the main findings?**

- RBW should be interpreted as an image-domain descriptor of processed transition width rather than as a measure of physical boundary-localization accuracy.
- Independent experimental repetitions, radiometric characterization, and traceable physical references are required before extending the results to quantitative engineering measurements.

**Abstract:**

Characterizing gas–liquid boundaries in underwater high-speed images is complicated by optical propagation and interface effects that broaden intensity transitions. Whether narrower transitions also indicate more accurate boundary localization, however, remains unclear. We integrated broadband white and narrow-band blue, green, and yellow source conditions with configuration-specific RBW processing at nominal observation distances of 0.7 and 5.0 m. Each source-distance condition was represented by one acquisition run, from which 20 temporally separated frames were analyzed as frame-level subsamples. In the 5.0 m run, the observed mean RBW was 17.46% under the blue-source condition and 36.18% under the white-source condition, corresponding to a descriptive reduction of 51.73%. A sensitivity analysis based on the interquartile range (IQR) yielded a descriptive reduction of 33.42%, whereas the near-field means were similar across source conditions. Target-plane optical irradiance and camera spectral responsivity were not calibrated. These values therefore describe source-condition responses of the complete imaging chain rather than isolated wavelength effects. A 12-image near-field holdout set annotated by two observers was used to compare the proposed estimator with classical and learning-based edge detectors. This image-reference benchmark did not support a claim of superior localization, and RBW showed little monotonic association with normalized image-reference localization discrepancy within the holdout set. The results characterize within-run image responses rather than reproducible source-condition effects. They support RBW as a descriptor of processed transition width, but not as a measure of physical boundary-localization accuracy.

## 1. Introduction

Multiphase flow is central to shipbuilding, marine navigation, and related marine engineering applications. High-speed optical imaging is widely used to capture transient gas–liquid interface dynamics [1], providing non-intrusive experimental data for validating computational fluid dynamics models. Accurate quantification of bubble morphology is also important for evaluating mass transfer, hydrodynamic efficiency, cavitation behavior, and underwater resource-exploration processes [2]. However, underwater imaging must resolve microsecond-scale interfacial motion across variable spatial scales, which imposes simultaneous requirements for high temporal resolution and reliable spatial boundary localization. High-fidelity underwater imaging has therefore become an important experimental tool in fluid mechanics [3,4]. In this context, reliable image-based delineation of gas–liquid interfaces provides an important basis for subsequent analyses of bubble morphology and dynamics.

The water column and the gas–liquid interface affect different stages of underwater image formation. During propagation, wavelength-dependent absorption and volumetric scattering attenuate the transmitted signal and contribute to the veiling background [5,6]. At the curved bubble interface, refraction, specular reflection, and possible total internal reflection redistribute the light entering the camera. Wavelength-dependent refraction and transmission through the water, observation window, surrounding air, and camera optics may additionally contribute to spectral focal spread [7]. Because the transfer functions of the individual optical components were not characterized, the present study treats these mechanisms as coupled system-level contributions. In the 0.7 m configuration, interface-induced intensity gaps and high-contrast background interference further complicated the sampled profiles [8,9]. Consequently, the recorded boundary transition reflects both distributed propagation degradation and local interface image formation.

Existing approaches to underwater interface imaging can be broadly grouped into image restoration and enhancement [10,11,12], rule-based or sensing-assisted interface extraction [13,14,15,16,17], and learning-based or computational imaging methods [18,19,20,21]. These methods primarily evaluate restored visibility, geometric contours, segmentation overlap, or object trajectories. Such endpoints do not determine whether a narrower processed intensity transition corresponds to closer agreement with the visible bubble contour. In the studies reviewed here, illumination-source conditions, observed profile morphology, transition-width characterization, and independent image-reference benchmarking are not evaluated within a single framework. The unresolved problem is therefore evaluative. Transition sharpness and boundary localization require separate characterization under the relevant acquisition conditions.

To address this gap, this study investigates gas–liquid boundary transitions under broadband white and narrow-band blue, green, and yellow illumination at two observation distances. The framework links the two acquisition configurations to their observed profile morphologies and applies separate one-dimensional branches to plateau-like near-field profiles and localized far-field responses. RBW is defined as an image-domain transition-width descriptor, while geometric localization is evaluated separately against a fused image-based reference. Accordingly, the benchmark evaluates image-domain agreement with a visible annotated contour rather than absolute physical interface accuracy. Comparator benchmarking, component ablation, and parameter-sensitivity analysis are used to identify both the utility and failure modes of the workflow. The individual operations are established; the contribution lies in their integration within an experimentally grounded characterization and evaluation framework, not in a new universal boundary-detection principle.

The remainder of this paper is organized as follows. Section 2 reviews relevant underwater imaging and gas–liquid interface-extraction methods and positions the present study within this literature. Section 3 describes the experimental configurations, image-processing procedures, image-reference benchmark, and statistical analyses. Section 4 presents the illumination comparisons, RBW results, boundary-localization benchmark, and ablation analysis. Section 5 discusses the optical interpretation, methodological scope, and distinction between transition width and localization accuracy. Section 6 summarizes the principal findings and limitations of the study.

## 2. Related Work

### 2.1. Underwater Image Restoration and Wavelength-Aware Optical Modeling

Physics-based underwater restoration methods model the wavelength-dependent absorption and scattering that produce color distortion, contrast loss, and veiling light. Wavelength-compensation and dehazing methods recover color and visibility by estimating differential attenuation among spectral channels [22], while multicolor attenuation models further describe the propagation differences among wavelength bands [23]. Related underwater optical studies have investigated scattering-aware LiDAR, polarization, and active imaging configurations [16,17,19]. These methods are primarily designed to restore scene radiance, improve perceptual image quality, or recover distant targets. They generally do not examine how broadband and narrow-band source conditions affect an image-domain gas–liquid boundary-transition metric within a fixed camera configuration.

### 2.2. Rule-Based Gas–Liquid Interface Extraction

Classical interface-extraction methods use intensity thresholds, gradients, watershed segmentation, background subtraction, and mathematical morphology to recover bubble or slug contours [13,14,15,21]. For example, do Amaral et al. combined watershed segmentation, Top-Hat filtering, and the H-minima transform to analyze bubbles in high-speed videos of horizontal two-phase slug flow [24]. These approaches are interpretable and computationally accessible, but their thresholds and structuring parameters can depend strongly on image contrast and background uniformity. Reflection artifacts, scattering pedestals, and spatially varying illumination may therefore be interpreted as interface features. The image-processing operations used here, including Top-Hat filtering, thresholding, Gaussian smoothing, and running-maxima envelope reconstruction, are established and are not claimed as methodological innovations. Their purpose is to construct profile representations suited to the observed morphologies in the two tested configurations within a common evaluation workflow.

### 2.3. Learning-Based Bubble and Interface Segmentation

Learning-based methods formulate interface extraction as object detection, semantic segmentation, or instance segmentation. Kim and Park trained Mask R-CNN for automated bubble detection and mask extraction in complex two-phase flows [25]. Homan and Deen reduced the manual-annotation burden by constructing synthetic bubble-swarm images from isolated experimental bubbles [26]. More recent studies have applied deep segmentation to boiling-bubble analysis [27] and combined YOLOv8 with optical flow for Taylor-bubble detection and velocity measurement [28]. These methods can recover two-dimensional masks or object trajectories, but they require annotated or synthetically generated training data and are evaluated using geometric detection or segmentation metrics. Their endpoints therefore differ from RBW, which describes the width of a sampled intensity transition.

Beyond task-specific bubble segmentation, Zhou et al. proposed multi-level region-wise knowledge distillation with prototype-balanced contrastive learning (MRKD-PBCL) for class-incremental semantic segmentation [29]. The method addresses catastrophic forgetting, background semantic drift, and class imbalance as new semantic classes are introduced. This approach is relevant to the future development of segmentation systems that must adapt to previously unseen bubble or flow regimes. However, MRKD-PBCL addresses model-level knowledge retention rather than underwater image formation, illumination-source configuration, or boundary-transition characterization. It is therefore discussed as an adjacent learning-based development rather than as a direct baseline for RBW.

### 2.4. Positioning of the Present Study

The present study is positioned as an experimental characterization and evaluation framework rather than as a fundamentally new edge detector. It links four illumination-source conditions, two acquisition configurations, configuration-specific one-dimensional profile processing, normalized RBW characterization, and an image-reference benchmark. This integration permits transition sharpness and image-reference localization discrepancy to be examined as distinct endpoints. The study therefore evaluates when the workflow is informative, but does not claim that its component operations constitute a new boundary-detection principle or that their combination is universally superior.

## 3. Materials and Methods

### 3.1. Illumination-Source and Imaging Configurations

**Figure 1:**
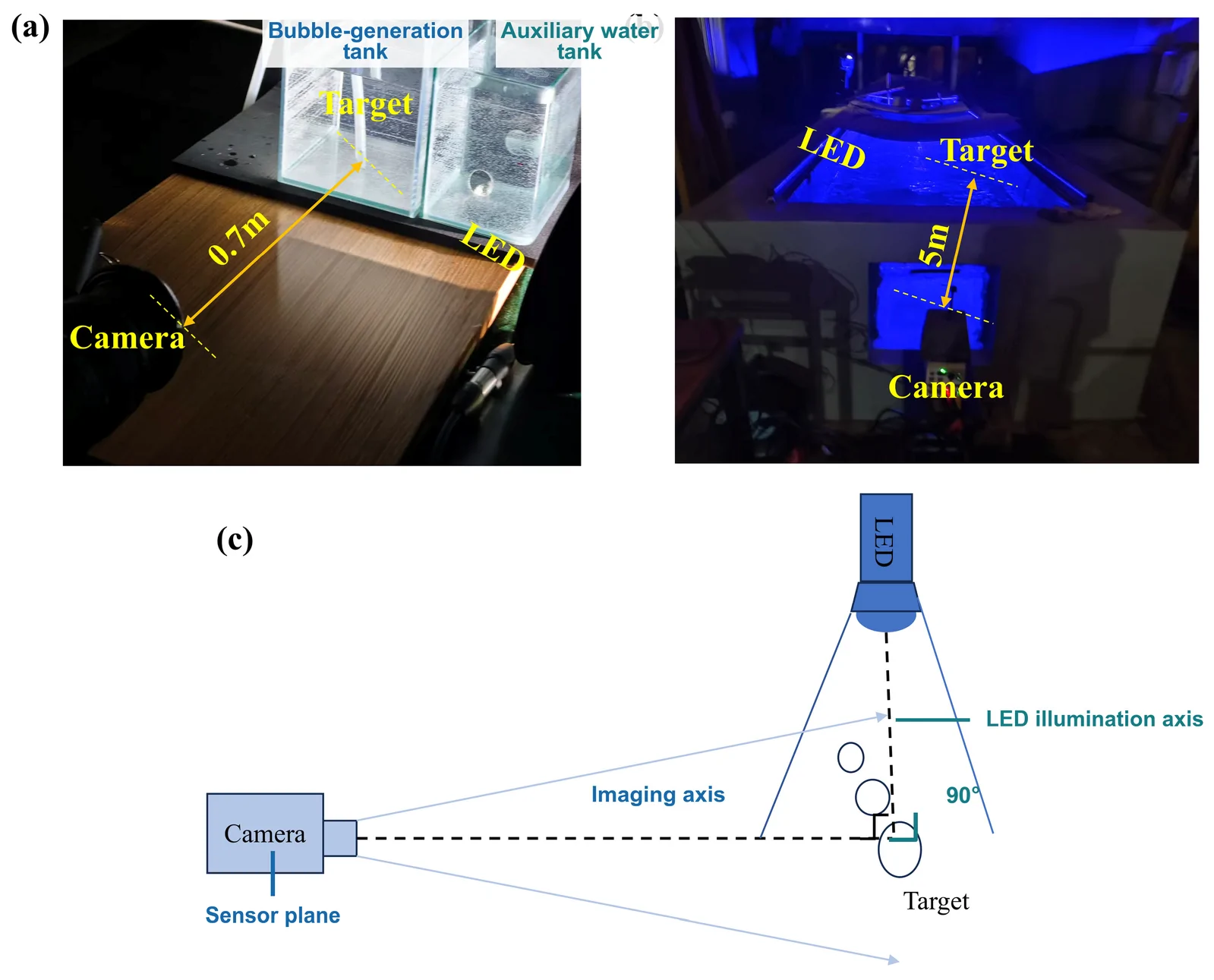
Experimental setup and optical configuration. (**a**) Near-field apparatus. The 0.7 m observation distance was measured along the camera optical axis from the sensor plane to the approximate bubble center. The auxiliary water-filled tank was positioned along the orthogonal LED illumination path and was not part of the camera-to-target imaging path. (**b**) Far-field apparatus, with a 5.0 m sensor-to-target observation distance and approximately 4.80 m of underwater propagation. (**c**) Top-view schematic showing the perpendicular imaging and illumination axes.

Figure 1 shows the near-field and far-field experimental configurations. In both configurations, the nominal observation distance was defined along the camera optical axis from the sensor plane of the high-speed camera to the approximate center of the imaged bubble. Near-field observations were conducted using a transparent rectangular bubble-generation tank measuring 23 × 16 × 18 cm, in which a capillary tube continuously generated ascending bubbles. In the 0.7 m configuration, the sensor-to-target distance comprised an approximately 0.20 m geometrical offset from the sensor plane to the front of the camera–lens assembly, 0.40 m of external propagation through air, and 0.10 m of propagation through water. The second tank visible in Figure 1a was an auxiliary water-filled tank positioned along the LED illumination path and was not part of the camera-to-target imaging path. Because a single tank spanning the required illumination distance was unavailable, the auxiliary tank was used to reduce propagation through air along the approximately 1.0 m LED-to-target path. The LED illumination axis was perpendicular to the camera optical axis, as illustrated in Figure 1c. Far-field observations were conducted in a 19 m water pool. The nominal sensor-to-target distance was 5.0 m, of which approximately 4.80 m corresponded to underwater propagation and approximately 0.20 m to the sensor-plane-to-lens-front geometrical offset.

Both configurations used glass observation windows. The camera optical axis was approximately normal to the respective observation window, whereas the orthogonal illumination beam entered through a separate glass wall. The glass composition, window thickness, illumination incidence angle, and wavelength-dependent window transmittance were not recorded. Because the enclosures and interface sequences differed, the two configurations should not be assumed to have identical optical transfer functions. No quantitative correction for window transmission was applied.

Bubble dynamics were recorded using a Revealer S1315 high-speed camera (HF Agile Device Co., Ltd., Hefei, China) fitted with a Canon EF 50 mm f/1.2L USM lens (Canon Inc., Tokyo, Japan). Images were acquired at 3000 frames per second with a resolution of 1280 × 1080 pixels. Automatic exposure was disabled, and the exposure time and gain were fixed at 328 μs and 1, respectively. The spectral responsivity of the camera was not independently calibrated. The aperture, focus position, depth of field, and chromatic-correction characteristics were not retained. Fixed exposure and gain ensured consistent camera settings but did not establish equivalent sensor exposure across the different source spectra. Consequently, the complete wavelength-dependent optical response could not be reconstructed retrospectively.

The light-emitting diode (LED) sources were positioned outside the enclosure and oriented perpendicular to the camera optical axis. The sources were assembled in-house from commercially sourced, nominally 100 W LED emitters whose manufacturer and model information was not retained. The DC supply voltage and current of each assembled source were measured using a SATA D05966 digital multimeter (SATA Tools (Shanghai) Co., Ltd., Shanghai, China), and the electrical input power was calculated as P=UI. The resulting electrical input powers during acquisition were 49.35 W for white, 49.45 W for blue, 48.58 W for green, and 48.64 W for yellow. The blue, green, and yellow sources had peak wavelengths of 435, 520, and 590 nm, respectively, with an approximate FWHM of 35 nm. These values characterize the electrical input and nominal spectral bands, not the optical radiant power incident on the bubble. No calibrated target-plane measurement of radiant power, irradiance, or spectral irradiance was performed, and the optical outputs were not radiometrically matched among sources. Similar electrical input powers therefore do not imply equivalent target illumination. The comparisons represent the complete response of each source–window–water–camera configuration rather than an isolated wavelength effect.

### 3.2. Configuration-Informed Dual-Track RBW Processing Workflow

**Figure 2:**
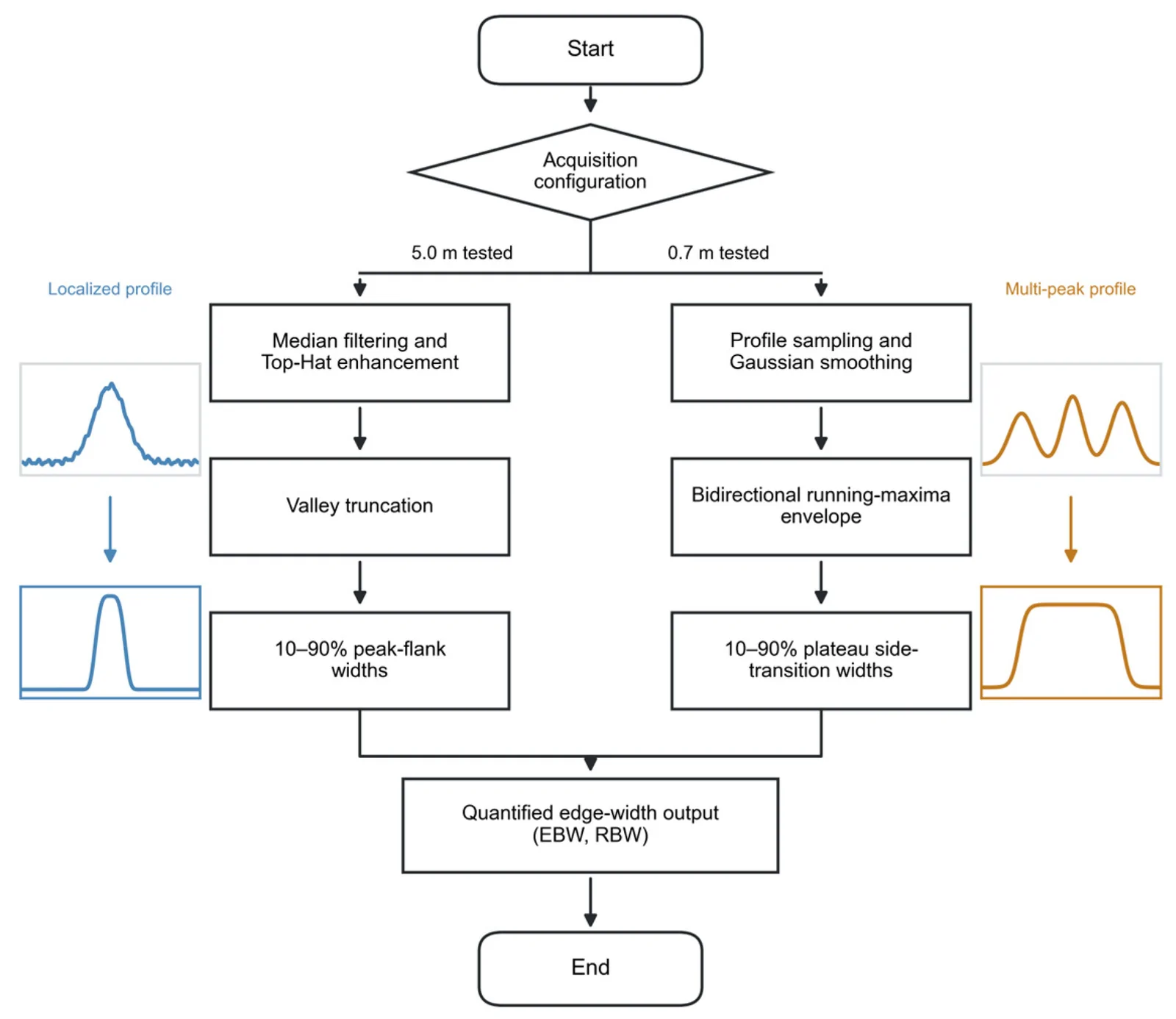
Configuration-informed dual-track workflow for RBW estimation. Images from the tested 5.0 m and 0.7 m configurations were assigned using acquisition metadata to the far-field (**left**) and near-field (**right**) branches, respectively. The far-field branch applies median filtering, Top-Hat enhancement, and valley truncation, whereas the near-field branch uses profile sampling, one-dimensional Gaussian smoothing, and bidirectional running-maxima envelope reconstruction. Both branches quantify the corresponding 10–90% transition widths and normalize EBW by the apparent target diameter to obtain RBW.

Figure 2 summarizes the configuration-informed dual-track workflow. Each ROI was assigned to a processing branch using the recorded acquisition metadata. Data acquired at the nominal 5.0 m camera-to-target distance were processed using the far-field branch, whereas data acquired at 0.7 m were processed using the near-field branch. The terms “far-field” and “near-field” are operational labels for the two tested configurations rather than optical regimes separated by a validated universal distance threshold. Intermediate observation distances were not examined.

The near-field branch was designed for profiles affected by internal intensity gaps, interface-induced reflection and refraction, and background-pipe interference. Five horizontal profile strips were sampled at 30%, 40%, 50%, 60%, and 70% of the ROI height. Pixel intensities were median-aggregated across each 30-pixel-high strip to obtain a one-dimensional horizontal profile. The profile was then smoothed using a nine-pixel one-dimensional Gaussian kernel. In the OpenCV implementation, the Gaussian standard deviation was derived internally from the kernel size because the standard-deviation parameter was set to zero. The smoothed profile was normalized before envelope reconstruction.

Let $\hat{I}\left(x\right)$ denote the normalized near-field profile. To bridge internal intensity gaps, two directional running maxima were calculated. The left-to-right running maximum $M_{L}\left(x\right)$ was defined as the maximum intensity observed from the beginning of the profile to coordinate x:

(1) $$M_{L}\left(x\right)=\underset{t\leq x}{\max}\hat{I}\left(t\right),$$

where t denotes the profile coordinate. The right-to-left running maximum $M_{R}\left(x\right)$ was defined analogously as the maximum intensity observed from coordinate x to the end of the profile:

(2) $$M_{R}\left(x\right)=\underset{t\geq x}{\max}\hat{I}\left(t\right),$$

The reconstructed envelope $E\left(x\right)$ was calculated as the pointwise minimum of the two directional running maxima:

(3) $$E\left(x\right)=\min\left(M_{L}\left(x\right),M_{R}\left(x\right)\right),$$

This operation bridged internal intensity depressions and produced a plateau-like representation while retaining the two outer transition regions. The plateau region was identified using a threshold of 0.90 times the envelope maximum. The left and right 10–90% transition widths were subsequently measured from the reconstructed envelope and averaged to obtain the profile-level EBW.

The far-field processing branch was designed for images affected by accumulated scattering, attenuation, and veiling background light. These effects generate a diffuse low-frequency pedestal beneath the localized boundary signal. A morphological Top-Hat transform was applied to enhance localized high-frequency boundary components and suppress slowly varying background intensity. The Top-Hat transform $T\left(A\right)$ is calculated through the following formula:

(4) $$T\left(A\right)=A-\left(A\circ B\right),$$

The symbol ∘ denotes the standard morphological opening operation. After detecting the far-field target, the algorithm sampled a horizontal band centered on its bounding box. The band half-height was set to 0.15 times the detected bounding-box width, with a minimum of three pixels. Pixel intensities were median-aggregated across the rows of this band to produce a one-dimensional horizontal profile. The dominant local maximum was identified within the target-centered search interval, and valley truncation isolated the corresponding peak from the surrounding low-frequency pedestal. The resulting peak represents the aggregated image-intensity response of the detected bubble candidate within the sampled central band. It does not represent the complete unresolved bubble or an independently verified physical interface. No Gaussian function was fitted to this profile.

Each processing branch produced a one-dimensional intensity profile $I\left(x\right)$. Before threshold-based width measurement, each local profile was min–max normalized to the range [0, 1]. This rescaling reduces sensitivity to overall intensity offsets and amplitudes but does not remove spatially varying background structure. The normalized profile $\hat{I}\left(x\right)$ was defined as follows:

(5) $$\hat{I}\left(x\right)=\frac{I\left(x\right)-I_{min}}{I_{max}-I_{min}},$$

The variables $I_{max}$ and $I_{min}$ represent the local maximum and minimum intensity values, respectively. EBW was defined as the spatial pixel distance between the 10% and 90% normalized intensity thresholds. To account for both sides of the boundary profile, EBW was calculated by averaging the left and right transition-zone widths.

(6) $$EBW=\frac{\left|x_{left,90\%}-x_{left,10\%}\left|+\right|x_{right,90\%}-x_{right,10\%}\right|}{2},$$

The variable x denotes the specific pixel coordinate at the corresponding threshold percentage. This formulation quantifies the 10–90% width of the processed one-dimensional intensity transition. A larger EBW indicates a broader image-domain transition in the sampled profile; however, EBW does not directly measure absolute physical boundary-localization uncertainty. Because EBW in pixels depends on the apparent target size, an apparent diameter proxy $D_{\mathrm{pixel}}$ was calculated from the detected bounding box:

(7) $$D_{pixel}=\frac{W_{bbox}+H_{bbox}}{2},$$

where $W_{bbox}\;$ and $H_{bbox}$ denote the width and height of the detected bounding box, respectively. To facilitate within-study comparisons across different apparent target sizes, RBW was defined by normalizing EBW to $D_{pixel}$:

(8) $$RBW=\frac{EBW}{D_{pixel}}\times 100\%,$$

RBW expresses the processed 10–90% transition width as a percentage of the apparent bubble diameter. A lower RBW denotes a narrower normalized intensity transition under the present acquisition and processing configuration. RBW is therefore used for within-study comparison of transition width and should not be interpreted as a universal image-quality metric or a direct measure of physical boundary-localization accuracy.

Although the same threshold convention and normalization equation were used in both processing branches, the operational meaning of the measured width was branch-specific. In the near-field branch, profile-level EBW was defined as the mean 10–90% transition width of the left and right sides of the envelope-reconstructed plateau. In the far-field branch, EBW was defined as the mean 10–90% flank width of the isolated central-band peak after pedestal suppression. Near-field and far-field RBW values are therefore dimensionless descriptors of processed image-domain transition width, but they are not physically interchangeable measurements of interface thickness.

### 3.3. Data Acquisition, ROI Selection, and Implementation Details

The independent experimental unit for source-condition comparisons was a complete acquisition run. Each source-distance condition was represented by one run. From each run, 20 valid frames were sampled at intervals of approximately 100–200 frames, yielding 160 frame-level subsamples across the eight conditions. Frames containing incomplete bubbles, severe overlap, or evident out-of-focus motion were excluded during prescreening. The camera frame rate, exposure time, gain, lens setting, and acquisition geometry were fixed across source conditions. Each LED was operated at the source-specific electrical input reported in Section 3.1; equivalent optical irradiance was not assumed. Temporal separation reduced short-range redundancy but did not create independent experimental replication. The sampled frames were therefore used to characterize within-run variation only.

The region of interest (ROI) was selected around each isolated bubble before boundary extraction. For near-field images, the ROI contained the complete visible bubble while retaining background-pipe interference. For far-field images, the ROI was centered on the detected target and included the surrounding diffuse pedestal. The same ROI-selection criteria were applied across illumination-source conditions. All 160 selected observations satisfied the predefined validity criteria for RBW processing and were retained in the primary analysis. The grouped IQR procedure was applied only as a sensitivity analysis and did not determine inclusion in the primary dataset.

For each near-field frame, horizontal profile regions were sampled at 30%, 40%, 50%, 60%, and 70% of the ROI height. Each profile was obtained by median aggregation across a 30-pixel-high strip. Profile-level EBW was calculated by averaging the left and right plateau-transition widths. The frame-level EBW was defined as the median of at least three valid profile-level measurements. For each far-field frame, one central horizontal band was sampled from the automatically detected target. Its EBW was calculated by averaging the left and right flank widths of the isolated localized peak. RBW was subsequently obtained by normalizing the resulting frame-level EBW by the apparent target diameter.

All image processing and statistical analyses were implemented in Python 3.14.5 using OpenCV 4.13.0, NumPy 2.4.6, and pandas 3.0.3. The baseline near-field settings were a 30-pixel profile-strip height, a nine-pixel one-dimensional Gaussian kernel, and a plateau threshold of 0.90. The baseline far-field settings were a five-pixel median filter, a 60 × 60-pixel elliptical Top-Hat element, a binary threshold of 15, and a 3 × 3-pixel elliptical opening element. Acquisition metadata assigned each ROI to the branch corresponding to the tested 0.7 or 5.0 m configuration. The implementation does not estimate target depth from image content and cannot assign different branches to targets at different depths within the same frame. Extension to mixed-depth scenes would require independent depth estimation or object-level classification validated at additional observation distances.

A one-factor-at-a-time sensitivity analysis was conducted for the principal tunable parameters in each processing branch. For the near-field branch, the Gaussian kernel was evaluated at 5, 9, and 13 pixels; the profile-strip height at 20, 30, and 40 pixels; and the plateau threshold at 0.85, 0.90, and 0.95. For the far-field branch, the median-filter kernel was evaluated at 3, 5, and 7 pixels; the Top-Hat element at 45, 60, and 75 pixels; the binary threshold at 10, 15, and 20; and the opening element at 1, 3, and 5 pixels. The central value of each range was the baseline setting. Each parameter was varied independently while all other settings remained fixed. Every setting was applied to all 80 frames in the corresponding branch. Sensitivity was assessed descriptively using valid prediction coverage, the percentage change in each source-specific mean RBW relative to baseline, and preservation of the source-condition ranking. No inferential tests were applied to these comparisons because only one acquisition run was available per condition.

Candidate bubble contours were screened using geometric constraints. Contours with areas smaller than 10 pixels or larger than 15% of the image area were excluded. The remaining contours were required to have an aspect ratio between 0.3 and 3.0 and a circularity greater than 0.2. When multiple candidates were detected, the target bubble was selected by maximizing the score defined as contour area multiplied by the square of circularity. The equivalent pixel diameter was calculated as the average of the bounding-box width and height. EBW was then normalized by this equivalent diameter to obtain RBW. Measurements with RBW values of 0% or less, values greater than 150%, or non-positive scale values were treated as invalid processing outputs. None of the 160 selected observations failed these criteria. To evaluate sensitivity to extreme observations, the 1.5 × IQR rule was subsequently applied within each illumination-distance condition. This procedure excluded eight observations and was evaluated separately rather than replacing the raw primary dataset.

RBW values were summarized using the mean, standard deviation, median, interquartile range, minimum, and maximum. For each narrow-band source condition, the difference in mean RBW relative to the white-source condition at the same observation distance was reported as a descriptive percentage change. No inferential tests were applied to these condition-level comparisons because each condition was represented by a single acquisition run. The grouped 1.5 × IQR analysis was retained solely to assess the sensitivity of the descriptive mean differences to extreme frame-level observations.

### 3.4. Image-Reference Benchmark and Ablation Protocol

To evaluate image-based boundary localization independently of RBW, a validation subset comprising 20 near-field images was independently annotated by two annotators. Before contour fusion, inter-annotator agreement was quantified using the Dice coefficient, intersection over union (IoU), average symmetric surface distance (ASSD), and the 95th-percentile Hausdorff distance (HD95). The images were divided into an eight-image development set and a 12-image holdout set before comparator parameter selection. Five horizontal profile regions were sampled at 30%, 40%, 50%, 60%, and 70% of the ROI height. At each profile region, the left and right intersections with the manually delineated outer contour were extracted for both annotators, and the arithmetic mean of the corresponding coordinates was used as the fused image-based reference. Profiles intersecting annotated occlusions or incompletely visible boundaries were excluded, leaving 73 eligible edge observations in the holdout set. The fused profile-level coordinates constitute an independent image-based reference rather than an absolute physical ground truth. Three evaluation levels were distinguished. RBW describes the normalized width of a processed image-intensity transition, whereas BLE and its summary statistics quantify discrepancy relative to the fused image-based reference. Neither quantity measures error relative to the true physical gas–liquid interface because no traceable physical reference was available.

The same ROIs and profile locations were used to evaluate the proposed near-field estimator, Canny, Otsu, Sobel, Prewitt, Laplacian of Gaussian (LoG), and DexiNED [30]. Comparator parameters were selected using only the development set and were subsequently fixed for holdout evaluation. Because the fused image-based reference represented the visible outer contour, the P90 endpoint of the reconstructed transition was used as the proposed outer-transition estimate. The P50 endpoint was retained as the transition center and was not interpreted as the outer boundary. An endpoint analysis comparing P10, P50, and P90 was included to document this distinction. Component ablation was performed by evaluating the raw profile, Gaussian smoothing alone, envelope reconstruction alone, and the complete envelope-plus-smoothing procedure.

Comparator configurations were selected on the eight-image development set by prioritizing prediction coverage, followed by image-balanced MAE and overall MAE. The locked Canny configuration used a three-pixel Gaussian blur, thresholds of 20 and 60, and nine-pixel profile smoothing. Otsu used no preblur or profile smoothing and a center offset of 0.08 times the ROI width. Sobel used a five-pixel gradient kernel without preblur or profile smoothing; Prewitt used no preblur and nine-pixel profile smoothing; and LoG used a three-pixel Gaussian preblur and nine-pixel profile smoothing. DexiNED was evaluated without additional profile smoothing. Candidate edges were searched between 0.10 and 0.42 times the ROI width from the profile center for all methods except Otsu, for which the lower bound was 0.05. All settings were locked before holdout evaluation.

For each eligible boundary observation i, the boundary localization error ${\mathrm{BLE}}_{i}$ was defined as ${\mathrm{BLE}}_{i}=\left|{\hat{x}}_{i}-x_{i}^{\mathrm{ref}}\right|$, where ${\hat{x}}_{i}$ and $x_{i}^{\mathrm{ref}}$ denote the predicted and fused reference coordinates, respectively. The normalized boundary localization error ${\mathrm{NBLE}}_{i}$ was calculated as ${\mathrm{NBLE}}_{i}=\frac{{\mathrm{BLE}}_{i}}{d_{i}}\times 100\%$, where $d_{i}$ denotes the reference profile diameter. The mean absolute error (MAE) was calculated as the arithmetic mean of the valid ${\mathrm{BLE}}_{i}$ values. Localization performance was summarized using MAE, median BLE, 95th-percentile BLE, mean NBLE, prediction coverage, and tolerance-based precision, recall, and F1 score. Accordingly, BLE, NBLE, and MAE are reported as image-reference discrepancies and not as estimates of absolute physical boundary error. For each method and ablation variant, image-level cluster bootstrap resampling with 5000 resamples was used to summarize MAE variability within the fixed holdout set. The 2.5th and 97.5th percentiles are reported as 95% bootstrap intervals. For the paired comparison between the proposed estimator and DexiNED, 10,000 image-level cluster-bootstrap resamples were applied to observations for which both methods returned valid predictions. These intervals describe resampling variability among the holdout images and should not be interpreted as uncertainty across independent experimental runs.

Consistent with the within-10-pixel analysis, a valid prediction located within 10 pixels of the corresponding reference was classified as a true positive. A valid prediction outside this tolerance was classified as a false positive and left the corresponding reference unmatched. Prediction failures therefore contributed to false negatives. Manually ineligible boundary observations were excluded before calculating these metrics. Because the proposed estimator returns sparse boundary coordinates at five profile heights rather than a dense closed contour, algorithm-level IoU, Dice, Hausdorff distance, and edge-continuity ratio were not calculated. The edge signal-to-noise ratio was also excluded from cross-method ranking because the evaluated methods do not generate a common signal representation.

Finally, the within-dataset relationship between RBW and image-reference localization discrepancy was examined. The RBW of each holdout image was paired with its mean normalized absolute localization error across valid P90 estimates, and the relationship was summarized using Spearman’s rank correlation coefficient. A sensitivity analysis used the image-level mean absolute error in pixels. The image, rather than each profile position or boundary side, was used as the summary unit. Because all holdout images originated from one experimental run, the coefficients were interpreted descriptively and no population-level hypothesis test was performed.

## 4. Experimental Results

### 4.1. Representative Bubble Images Under the Tested Configurations

**Figure 3:**
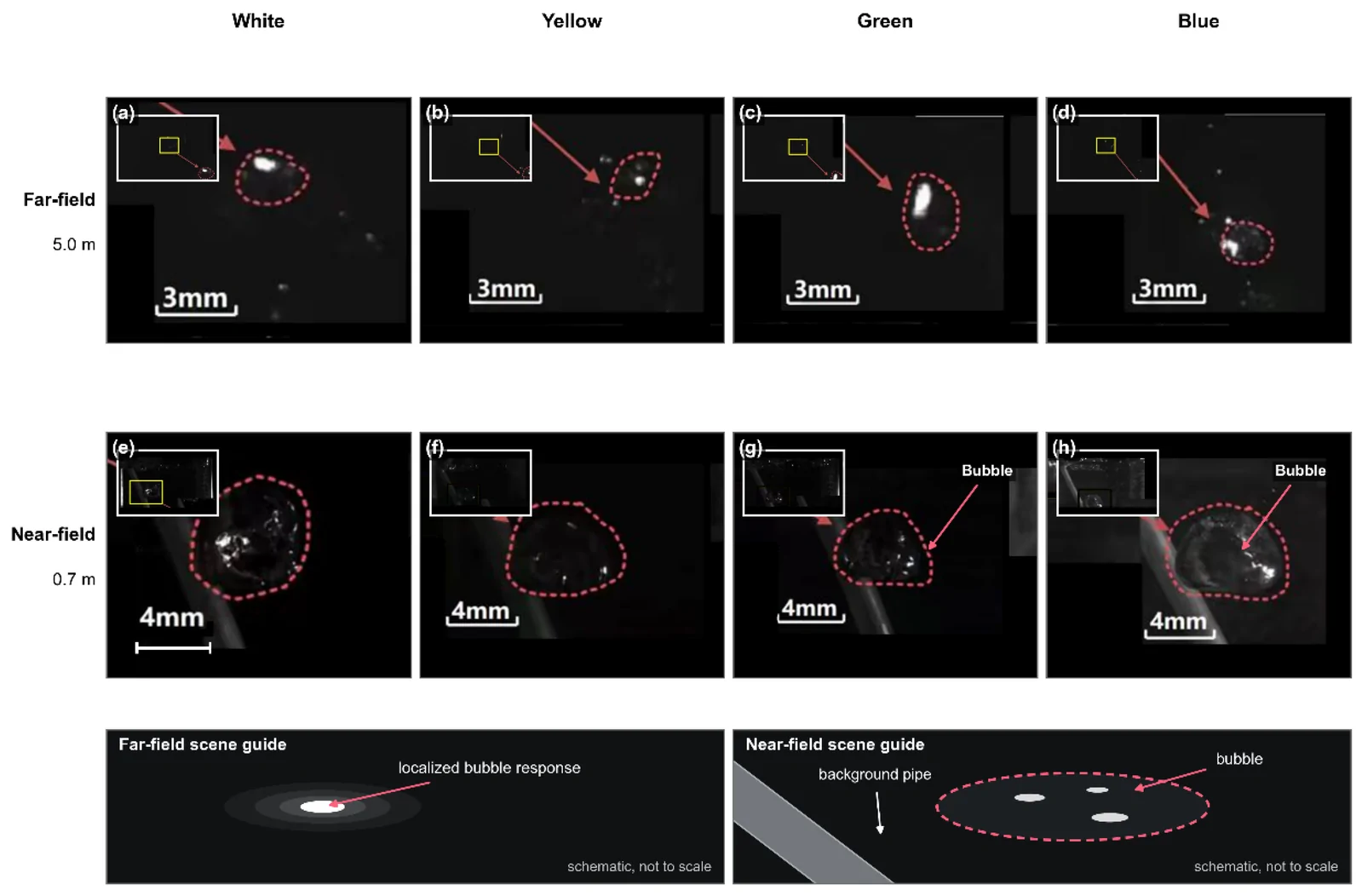
Representative bubble images under white, yellow, green, and blue illumination (left to right) in the 5.0 m (**a**–**d**) and 0.7 m (**e**–**h**) configurations. Each panel shows an enlarged ROI and an overview inset of the original frame; the yellow rectangle in each inset indicates the ROI displayed in the corresponding main panel. Pink outlines, arrows, and lower scene guides identify the bubble response and principal scene elements, including the oblique background pipe; these annotations are schematic, not quantitative contours, and were not used in the analysis. Scale bars: 3 mm (**a**–**d**) and 4 mm (**e**–**h**). No local brightness or contrast adjustment was applied.

Figure 3 presents representative bubble images acquired under white, yellow, green, and blue illumination in the 5.0 and 0.7 m configurations. To improve the visibility of the target regions, the enlarged ROI is presented as the main view in each panel, while the inset indicates the location of the ROI within the original field of view.

In the 5.0 m configuration (Figure 3a–d), images recorded under the broadband white-source condition exhibited a diffuse veiling background and a broader apparent boundary response. Images recorded under the blue-source condition showed the most compact visible boundary response among the four tested sources. These observations describe the output of the complete acquisition system and do not isolate the contribution of wavelength, target irradiance, or camera spectral responsivity. The far-field branch therefore applied Top-Hat filtering and valley truncation to suppress the observed low-frequency pedestal and isolate the dominant localized response.

In the 0.7 m configuration (Figure 3e–h), the bubbles appear as larger transparent regions containing multiple internal bright features. The oblique bright structure crossing the near-field panels is the background pipe rather than an additional bubble boundary. This overlap, together with interface-induced reflection and refraction, makes the bubble perimeter difficult to distinguish in the unannotated images. The pink dashed outlines are included only to assist visual interpretation and should not be regarded as algorithm outputs or physical interface measurements.

### 4.2. Configuration-Specific One-Dimensional Profile Transformations

**Figure 4:**
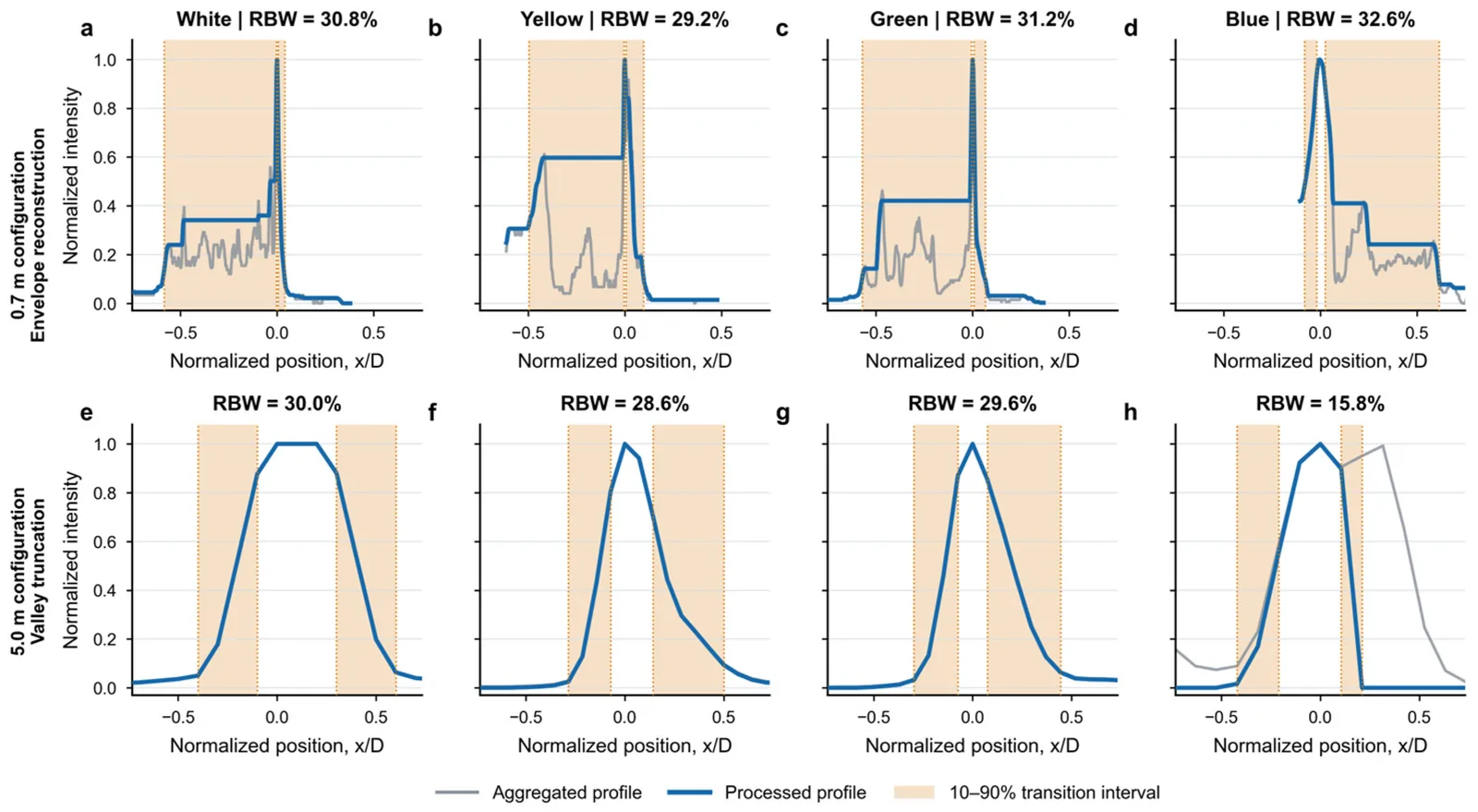
Representative one-dimensional profile transformations at 0.7 m (**a**–**d**) and 5.0 m (**e**–**h**) for the white, yellow, green, and blue source conditions. Each displayed frame had the RBW closest to its within-condition median. Gray curves show median-aggregated profiles, and blue curves show envelope-reconstructed (**a**–**d**) or valley-truncated (**e**–**h**) profiles. Orange regions and dotted bounds mark the left and right 10–90% transition intervals used for EBW calculation. Horizontal position is normalized by the apparent target diameter D, and the titles report frame-level RBW. These examples illustrate the processing and measurement definitions, whereas group-level comparisons are presented in Figure 5 and Table 1.

Figure 4 visualizes the profile transformations applied to one representative frame from each illumination–configuration condition. Each representative frame was selected as the observation with the RBW closest to the within-condition median. At 0.7 m (Figure 4a–d), median aggregation retained local intensity fluctuations, whereas the bidirectional running-maxima envelope produced the plateau-like profiles used for transition-width measurement. The displayed strip was the valid profile region with an EBW closest to the frame-level median EBW. The corresponding frame-level RBW values were 30.8%, 29.2%, 31.2%, and 32.6% under white, yellow, green, and blue illumination, respectively.

**Figure 5:**
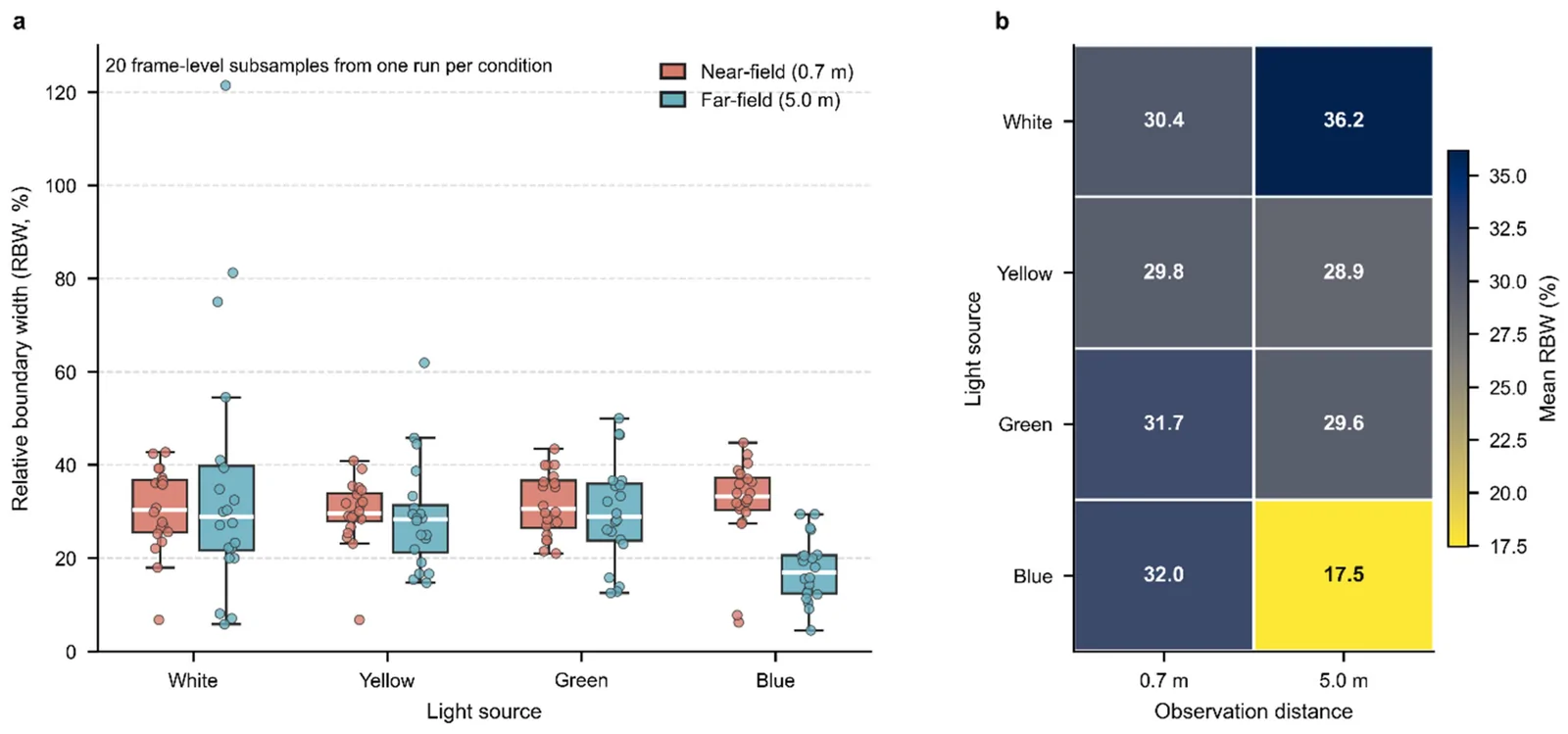
Descriptive frame-level distribution of relative boundary width (RBW). (**a**) Boxplots and individual frame-level subsamples for the 0.7 and 5.0 m configurations. Each dot represents the RBW value obtained from one frame-level subsample; slight horizontal jitter was applied only to reduce visual overlap. Each condition contains 20 temporally separated frames sampled from one acquisition run. Boxes indicate the median and interquartile range, and whiskers extend to 1.5 times the interquartile range. (**b**) Raw descriptive mean RBW for each source-distance condition. The panels characterize within-run variation and do not represent independent experimental replication or between-run inference.

At 5.0 m (Figure 4e–h), valley truncation isolated the localized profile response and suppressed the signal outside the detected valley limits. The shaded regions mark the 10–90% transition intervals on the left and right flanks. The representative frame-level RBW values were 30.0%, 28.6%, 29.6%, and 15.8% under white, yellow, green, and blue illumination, respectively. These profiles illustrate the configuration-specific processing and measurement procedures. Descriptive source-condition summaries are presented separately in Figure 5 and Table 1. Accordingly, the valley-truncated far-field peak represents the aggregated central-band image response rather than the complete unresolved bubble or a directly measured physical interface.

### 4.3. Quantitative Analysis of Relative Boundary Width

Figure 5a presents the 20 frame-level RBW subsamples obtained from the single acquisition run available for each source-distance condition, whereas Figure 5b reports their raw descriptive means. All frames meeting the predefined RBW-processing validity criteria were retained. The distributions characterize within-run variation, and no inferential comparison among source conditions was performed.

Because mean values alone can obscure frame-level variability, Table 1 reports the mean and standard deviation, median and interquartile range, range, and descriptive mean change relative to the white-source condition. The primary summaries include all 20 frame-level subsamples per condition. Complete frame-level EBW and RBW records are provided in Data S1, while the IQR-filtered results are reported as a sensitivity analysis.

In the 0.7 m configuration, the observed mean RBW values were 30.42% for white, 31.95% for blue, 31.66% for green, and 29.75% for yellow source conditions. Relative to the white-source mean, the descriptive changes were −5.02%, −4.05%, and 2.21% for blue, green, and yellow, respectively. These small and inconsistent within-run differences did not identify a stable near-field source-condition advantage.

In the 5.0 m configuration, the blue-source condition had the lowest observed mean RBW at 17.46%, compared with 36.18% for the white-source condition. This corresponded to a descriptive within-run reduction of 51.73%. Green and yellow had observed mean RBW values of 29.64% and 28.88%, corresponding to descriptive reductions of 18.08% and 20.16%, respectively.

The 5.0 m white-source distribution was right-skewed and included a maximum RBW of 121.43%. The IQR sensitivity analysis excluded eight frames overall, including three white-source frames at 5.0 m. The resulting white-source mean was 26.23%, while the blue-source mean remained 17.46%. The descriptive reduction was therefore 33.42%. The direction of the observed contrast was retained, although its magnitude was sensitive to extreme white-source observations.

### 4.4. Image-Reference Comparison and Ablation Analysis

Before evaluating the boundary estimators, inter-annotator agreement was assessed across the 20 manually annotated near-field images. The two annotators achieved a mean Dice coefficient of 0.945, a mean intersection over union (IoU) of 0.897, a mean average symmetric surface distance (ASSD) of 5.49 pixels, and a mean 95th-percentile Hausdorff distance (HD95) of 14.44 pixels. These metrics indicate substantial repeatability between the two image-based annotations. They do not establish the accuracy of either annotation relative to the physical gas–liquid interface. The complete annotation overlays are provided in Supplementary Figure S1.

The fused annotations were subsequently used as the image-based reference for the holdout comparison. Table 2 compares the proposed P90 outer-transition estimate with the conventional and learning-based detectors. DexiNED achieved the lowest overall MAE of 21.34 pixels, followed by Prewitt and Sobel. The proposed estimator yielded an MAE of 36.05 pixels, a median absolute error of 15.25 pixels, and 94.5% prediction coverage. Its relatively low median error and 39.1% within-10-pixel rate indicate that many estimates were close to the image-based reference; however, the P95 error of 112.55 pixels reveals a substantial failure tail. Of the 69 observations returned by both the proposed estimator and DexiNED, their respective MAEs were 36.05 and 21.05 pixels, producing a paired difference of 15.00 pixels (95% image-level cluster-bootstrap interval of 3.45–24.25 pixels). This interval describes variability within the fixed holdout dataset rather than uncertainty across independent experimental runs. Thus, the holdout results do not support a claim of superior outer-boundary localization.

To incorporate prediction failures into the evaluation, tolerance-based precision, recall, and F1 score were additionally calculated for all seven methods. At the 10-pixel tolerance, the proposed estimator achieved a precision of 0.391, a recall of 0.370, and an F1 score of 0.380. DexiNED achieved corresponding values of 0.342, 0.342, and 0.342. Although the proposed estimator returned a greater proportion of estimates within the specified tolerance, DexiNED produced substantially lower mean and tail localization errors. The complete multi-metric results are provided in Supplementary Table S1.

The endpoint analysis showed that the MAE decreased from 88.94 pixels at P10 and 57.61 pixels at P50 to 36.05 pixels at P90. This result indicates that, under the present validation protocol, the outer-transition endpoint was more appropriate than the transition center for comparison with the annotated outer contour. In the component ablation, the raw-profile and smoothing-only variants produced MAEs of 56.29 and 55.37 pixels, respectively. Envelope reconstruction reduced the MAE to 36.75 pixels, whereas subsequent Gaussian smoothing produced only a small further reduction to 36.05 pixels. The largest observed reduction was therefore associated with envelope reconstruction. Nevertheless, the remaining high-error cases limit the reliability of the proposed estimator as a general-purpose contour-localization method.

The parameter sensitivity analysis retained 100% valid prediction coverage for every evaluated setting (Supplementary Figure S2 and Data S2). In the near-field branch, the maximum absolute change in a source-specific mean RBW was 5.86%. Reducing the profile-strip height from 30 to 20 pixels changed the source condition with the lowest mean RBW from yellow to white, indicating that the near-field ranking was not stable. In the far-field branch, the blue-source condition retained the lowest observed mean RBW under every parameter perturbation. Its descriptive mean reduction relative to the white-source condition ranged from 27.97% to 53.10%. The source ranking was therefore retained across the tested settings, although the absolute RBW values and effect magnitude remained parameter-dependent.

### 4.5. Association Between RBW and Image-Reference Localization Discrepancy

The preceding analysis evaluated outer-contour localization independently of RBW. Across the 12 holdout images, the descriptive Spearman coefficient between RBW and mean normalized localization discrepancy was −0.126. The sensitivity analysis using mean absolute error in pixels yielded a coefficient of −0.210. These values indicated little monotonic correspondence within the fixed holdout dataset, but they cannot be generalized across independent experimental runs.

Individual-image results further illustrated this weak correspondence. For example, NF007 had an RBW of 6.25% but a mean image-reference localization discrepancy of 49.65 pixels, whereas NF011 had an RBW of 37.23% but a corresponding discrepancy of 5.60 pixels. Thus, within this holdout set, a narrower processed intensity transition did not necessarily correspond to an outer-transition estimate closer to the annotated contour. Given the limited holdout set and its origin from one experimental run, this weak within-dataset correspondence should not be generalized to other acquisitions.

## 5. Discussion

### 5.1. System-Level Interpretation of Source-Condition Differences at 5.0 m

**Figure 6:**
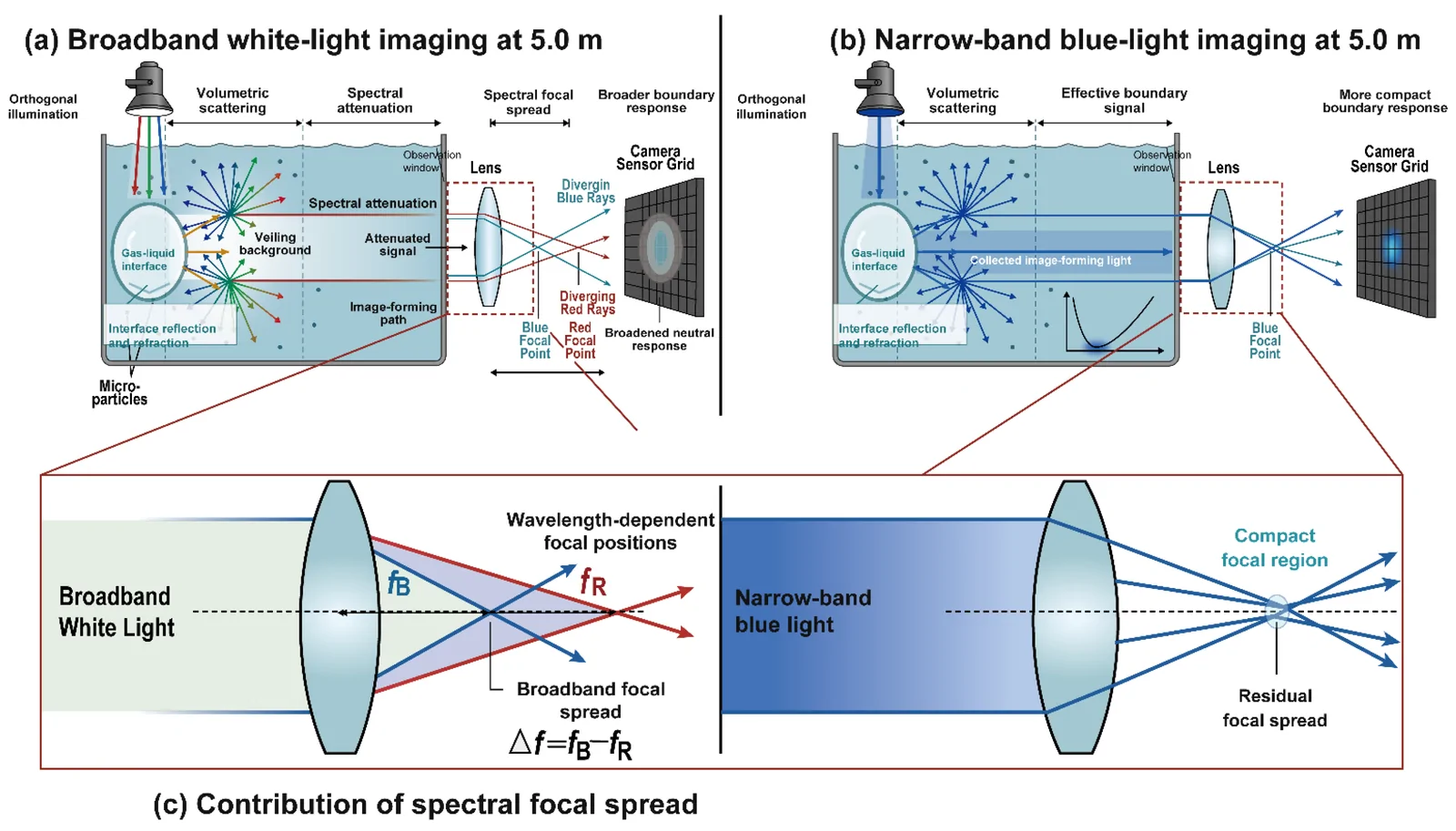
Qualitative schematic of candidate optical mechanisms in the tested 5.0 m configuration. Panels (**a**,**b**) distinguish orthogonal LED illumination from image-forming light collected through the observation window along the camera axis and separate distributed attenuation, volumetric scattering, and veiling background from reflection and refraction at the curved gas–liquid interface. In panel (**a**), multicolored arrows denote representative broadband spectral components and their scattering or image-forming paths; in panel (**b**), blue and cyan arrows denote narrow-band blue-light paths, while black arrows indicate general propagation or process direction. Panel (**c**) uses red and blue rays to illustrate wavelength-dependent focal positions and spectral focal spread as one possible contribution to boundary broadening. Arrow colors are schematic and do not encode calibrated radiometric magnitudes. The diagram does not represent radiometrically matched source conditions or a calibrated optical-transfer model, does not quantify the contributions of individual mechanisms, and is not drawn to scale.

The observed differences among the 5.0 m source conditions must be interpreted at the system level. Although the four LED modules had similar electrical input powers, their optical outputs at the bubble were not measured or radiometrically matched. Window transmission and camera spectral responsivity were also not independently characterized. The recorded differences may therefore reflect source-specific radiant output, emitted spectrum, window transmission, water-column propagation, interface image formation, and camera response. The present data cannot separate these contributions or estimate an isolated causal effect of illumination wavelength. Figure 6 provides a qualitative schematic of the candidate mechanisms considered in this system-level interpretation.

The recorded boundary image reflects two physically distinct optical stages. Along the illumination and image-forming paths, absorption, volumetric scattering, and veiling-background formation affect signal propagation. At the bubble, refraction, specular reflection, and possible total internal reflection redistribute the light collected by the camera. For the millimetric bubbles examined here, these interface effects are more appropriately interpreted using geometric optics than by treating the bubble itself as a Rayleigh scatterer [31].

Rayleigh scattering increases at shorter wavelengths [5]. The lower observed RBW under the blue-source condition therefore cannot be attributed to inherently weaker Rayleigh scattering. Within the sampled 5.0 m run, the blue-source condition had the lowest mean RBW. This pattern may reflect the combined influence of source output, absorption, volumetric scattering, interface-induced light redistribution, spectral spread, optical transmission, sensor responsivity, and residual boundary contrast. Broadband white illumination spans a wider spectral range and may contribute to a broader system response, whereas narrow-band illumination restricts the incident spectral range. These mechanisms provide a qualitative interpretation of the observed profile morphology but do not establish a universal wavelength optimum.

### 5.2. Spectral Focal Spread as One Component of Boundary Broadening

Spectral focal spread represents one component of the complete imaging-chain response rather than an effect attributable solely to the camera lens. Broadband radiation undergoes wavelength-dependent refraction and transmission through the water–window–air path and the lens assembly. These combined effects may produce wavelength-dependent focal positions at the sensor. The separation between representative blue and red focal positions is expressed as $\Delta f=f_{B}-f_{R}$, where $f_{B}$ and $f_{R}$ denote the representative focal positions of the blue and red spectral components, respectively. Because the window and lens transfer characteristics were not independently characterized, the contribution of each optical element cannot be determined from the present data. Figure 6c therefore provides a qualitative illustration of spectral focal spread rather than a calibrated model of the complete optical transfer function.

Narrow-band illumination limits the spectral range entering the imaging chain and may therefore reduce one component of spectral focal spread. It does not eliminate optical blur because the LEDs have finite bandwidths and the recorded response is also affected by source output, water-column propagation, window transmission, interface reflection and refraction, lens aberrations, diffraction, sensor sampling, and camera spectral responsivity. The more compact response observed under the blue-source condition may reflect the combined response of these factors. Without radiometric and spectral calibration, the relative contribution of source wavelength or bandwidth cannot be determined.

### 5.3. Coupling Between Optical Mechanism and Dual-Track Edge Extraction

The two processing branches should be interpreted as configuration-specific responses to the profile morphologies observed in the two tested experiments, rather than as optical regimes separated by a universal distance threshold. In the 5.0 m configuration, the boundary response was superimposed on a broadened low-frequency pedestal associated with accumulated attenuation, volumetric scattering, veiling background, and residual spectral blur. Top-Hat enhancement and valley truncation were therefore used to isolate the localized response before far-field EBW and RBW estimation. In the 0.7 m configuration, the observed profiles were dominated by internal intensity gaps, interface-induced reflection and refraction, and background-pipe interference. The bidirectional running-maxima envelope was used to reconstruct a plateau-like representation before measuring its side-transition widths. These operations correspond to the profile degradation patterns observed in the two tested configurations, but the present data do not identify the distance at which one pattern becomes dominant.

Branch assignment currently relies on acquisition metadata and assumes that each processed ROI belongs to one known configuration. The workflow does not infer depth from image content and cannot jointly process targets belonging to different depth regimes within the same frame. Application to mixed-depth scenes would require object-level depth or optical-regime estimation and validation using additional intermediate-distance data.

### 5.4. Methodological Contribution and Scope of RBW

The methodological contribution of this study lies at the workflow and evaluation levels rather than in a new elementary edge-detection operation. The framework links illumination-source conditions and acquisition configurations to branch-specific profile representations and evaluates the resulting transition characteristics using RBW and a separate image-reference benchmark.

Although the two processing branches use the same 10–90% threshold convention and diameter normalization, their RBW values are not physically interchangeable. Near-field RBW characterizes the transition slopes at the sides of an envelope-reconstructed plateau, whereas far-field RBW characterizes the flanks of a localized central-band response after pedestal suppression. Direct comparison of their absolute values would therefore confound profile morphology, sampling strategy, apparent target size, and acquisition configuration. The appropriate interpretation is a source-condition comparison within each processing branch under a fixed acquisition geometry.

RBW and BLE describe different image-domain properties. RBW quantifies the 10–90% intensity-transition width relative to the apparent bubble diameter, whereas BLE quantifies the coordinate discrepancy between a selected transition endpoint and the fused image-based reference. A narrow transition may therefore occur at an image feature displaced from the annotated outer contour. Conversely, a broader transition may still contain an endpoint close to that contour.

Consistent with this distinction, little monotonic association between RBW and image-reference localization discrepancy was observed in the fixed holdout set. This observation does not invalidate RBW as a descriptor of transition sharpness; instead, it limits the conclusions that can be drawn from RBW alone. The source-condition summaries characterize processed image-domain transition width under the tested acquisition configurations and should not be interpreted as evidence of improved physical boundary localization. Establishing physical measurement accuracy would require an independently calibrated boundary target or another traceable physical reference.

Within this defined scope, the workflow provides a controlled means of comparing image-formation and processing choices for transition characterization under fixed acquisition configurations. It does not validate the physical interface position or downstream quantities such as bubble diameter, projected area, volume, velocity, or energy dissipation. Quantitative multiphase-flow measurement would require traceable geometric calibration and independent validation of the physical interface.

The expanded frame-level dataset characterized within-run variation but did not increase the number of independent experimental repetitions. Frames within the same condition shared run-level acquisition and environmental factors, and temporal separation did not remove this dependence. Source-condition rankings and percentage reductions are therefore reported descriptively, without inferential condition-level tests. These patterns may be specific to the sampled runs. Independent experimental repetitions and run-level or hierarchical analyses are required to assess reproducibility across acquisitions.

The multi-metric benchmark further shows that localization performance cannot be represented by a single statistic. The proposed estimator had the highest observed F1 score at the 10-pixel tolerance, whereas DexiNED produced substantially lower mean and tail BLE. This combination indicates that the proposed procedure frequently returned estimates near the annotated contour but remained susceptible to large errors in some profiles. It should therefore be interpreted as a configuration-specific transition-characterization procedure rather than a generally superior two-dimensional contour-localization method.

## 6. Conclusions

This study developed and evaluated an integrated workflow for characterizing processed gas–liquid boundary transitions under four illumination-source conditions at 0.7 and 5.0 m. The configuration-informed dual-track workflow reconstructed plateau-side transitions in the 0.7 m images and isolated localized peak flanks in the 5.0 m images. Its principal contribution is the integration of acquisition, configuration-specific profile processing, RBW characterization, and image-reference benchmarking. The framework distinguishes image-domain transition sharpness from agreement with an annotated outer contour and is not presented as a new universal boundary detector. Because target-plane irradiance, window transmission, and camera spectral responsivity were not calibrated, the observed source-condition differences represent responses of the complete acquisition system and cannot be attributed to illumination wavelength alone.

Across 20 frame-level subsamples drawn from one run per condition, the 5.0 m blue-source run had an observed mean RBW of 17.46%, compared with 36.18% in the white-source run. The raw descriptive reduction was 51.73%, while the IQR sensitivity analysis yielded 33.42%. Near-field mean RBW values were similar across source conditions. These results characterize within-run image responses and do not establish reproducible condition-level effects. In the fixed 12-image holdout set, the proposed P90 estimator did not support a claim of overall localization superiority, and RBW showed little monotonic association with normalized image-reference localization discrepancy. RBW should therefore be interpreted as a descriptor of processed image-domain transition width rather than as a measure of physical boundary-localization accuracy.

The conclusions are limited to static water, room temperature, atmospheric pressure, two-dimensional imaging, a finite bubble-size range, two acquisition distances, and one experimental run per condition. No physical interface location or downstream engineering quantity, including bubble diameter, projected area, volume, velocity, or energy dissipation, was independently validated. Branch assignment relied on acquisition metadata and cannot currently accommodate mixed-depth targets within one frame. Independent experimental repetitions, target-plane radiometric calibration, characterization of window transmission and camera spectral responsivity, intermediate observation distances, and a traceable physical boundary reference are required before extending the framework to quantitative engineering measurements. Future validation could use a submerged calibration target with known edge geometry, an optical bubble phantom, or synchronized multi-view measurements linked to an independent physical reference.

## Figures and Tables

**Table 1:** Descriptive within-run summary of frame-level RBW by illumination-source condition and observation configuration.

Configuration	Light Source	Independent Runs	Frame Subsamples	Mean (SD), %	Median (IQR), %	Min–Max, %	Mean RBW Reduction vs. White, %
Near-field (0.7 m)	White	1	20	30.42 (9.01)	30.37 (11.25)	6.80–42.75	Reference
Near-field (0.7 m)	Blue	1	20	31.95 (9.69)	33.29 (6.85)	6.25–44.77	−5.02
Near-field (0.7 m)	Green	1	20	31.66 (6.85)	30.58 (10.25)	21.02–43.50	−4.05
Near-field (0.7 m)	Yellow	1	20	29.75 (7.11)	29.61 (5.92)	6.73–40.88	2.21
Far-field (5.0 m)	White	1	20	36.18 (28.16)	28.80 (18.14)	5.81–121.43	Reference
Far-field (5.0 m)	Blue	1	20	17.46 (6.86)	16.92 (8.20)	4.49–29.41	51.73
Far-field (5.0 m)	Green	1	20	29.64 (11.00)	28.92 (12.18)	12.50–50.00	18.08
Far-field (5.0 m)	Yellow	1	20	28.88 (11.74)	28.29 (10.24)	14.71–61.90	20.16

Note: The independent experimental unit was a complete acquisition run. Each source-distance condition comprised one run, from which 20 temporally separated frames were analyzed as frame-level subsamples. The table therefore reports descriptive within-run summaries and does not support condition-level statistical inference. Positive reduction values indicate a lower mean RBW than the white-source condition at the same observation distance, whereas negative values indicate a higher mean RBW. Near-field and far-field RBW values were derived from different profile morphologies and are not physically interchangeable.

**Table 2:** Image-reference comparison and component ablation on the 12-image near-field holdout set.

Analysis	Method or Variant	MAE, Pixels (95% Bootstrap Interval)	Median BLE, Pixels	P95 BLE, Pixels	Within 10 px, %	Coverage
Comparison	Proposed P90 estimator	36.05 (27.58–43.71)	15.25	112.55	39.1	94.5%
Comparison	Canny	25.08 (18.17–32.42)	22.50	70.00	29.6	97.3%
Comparison	Otsu	39.76 (26.61–50.44)	27.25	118.70	18.8	94.5%
Comparison	Sobel	23.17 (17.74–27.54)	22.50	56.60	31.5	100.0%
Comparison	Prewitt	22.03 (16.46–26.53)	22.50	56.20	32.9	100.0%
Comparison	LoG	26.68 (19.89–32.44)	23.75	65.75	27.4	100.0%
Comparison	DexiNED	21.34 (15.72–26.69)	20.50	47.90	34.2	100.0%
Ablation	Raw profile	56.29 (34.34–75.23)	23.00	180.00	29.6	97.3%
Ablation	Gaussian smoothing only	55.37 (33.81–75.70)	23.00	180.80	30.4	94.5%
Ablation	Envelope reconstruction only	36.75 (27.51–44.74)	15.50	114.88	39.4	97.3%
Ablation	Envelope plus smoothing	36.05 (27.58–43.71)	15.25	112.55	39.1	94.5%

Note: MAE and “Within 10 px” values were calculated from valid predictions only; coverage must therefore be considered when comparing methods. “Within 10 px” denotes the percentage of valid predictions located within 10 pixels of the fused image-based reference and should not be interpreted as recall. The 95% bootstrap intervals summarize image-level resampling variability within the 12-image holdout set and do not represent uncertainty across independent experimental runs.

## Data Availability

The processed frame-level RBW records and parameter-sensitivity results are provided in the Supplementary Materials as Data S1 and Data S2. The raw high-speed image sequences and analysis code are available from the corresponding author upon reasonable request.

## Supplementary Materials

The following supporting information can be downloaded at: https://www.mdpi.com/article/10.3390/s26175546/s1, Figure S1, independent manual annotations of the 20 near-field validation images; Figure S2, one-factor-at-a-time sensitivity analysis of the RBW-processing parameters; Table S1, multi-metric profile-level boundary-localization comparison on the 12-image near-field holdout set; Data S1, complete frame-level RBW-processing records; Data S2, parameter-sensitivity results.

## Abbreviations

The following abbreviations are used in this manuscript:

ASSD	Average symmetric surface distance
BLE	Boundary localization error
EBW	Edge boundary width
FWHM	Full width at half maximum
HD95	95th-percentile Hausdorff distance
IoU	Intersection over union
IQR	Interquartile range
LED	Light-emitting diode
LoG	Laplacian of Gaussian
MAE	Mean absolute error
NBLE	Normalized boundary localization error
RBW	Relative boundary width
ROI	Region of interest
SD	Standard deviation
