# Supplementary material for: Boundary-Transition Characterization in High-Speed Underwater Bubble Imaging Under Broadband and Narrow-Band Illumination
Source: Sensors (Basel). 2026 Aug 31;26(17):5546. doi: 10.3390/s26175546 (PMC13568190; doi:10.3390/s26175546)
Supplement: Supplementary file 1 [file sensors-26-05546-s001.zip › Supplementary File-Figure S1-round 2.pdf]

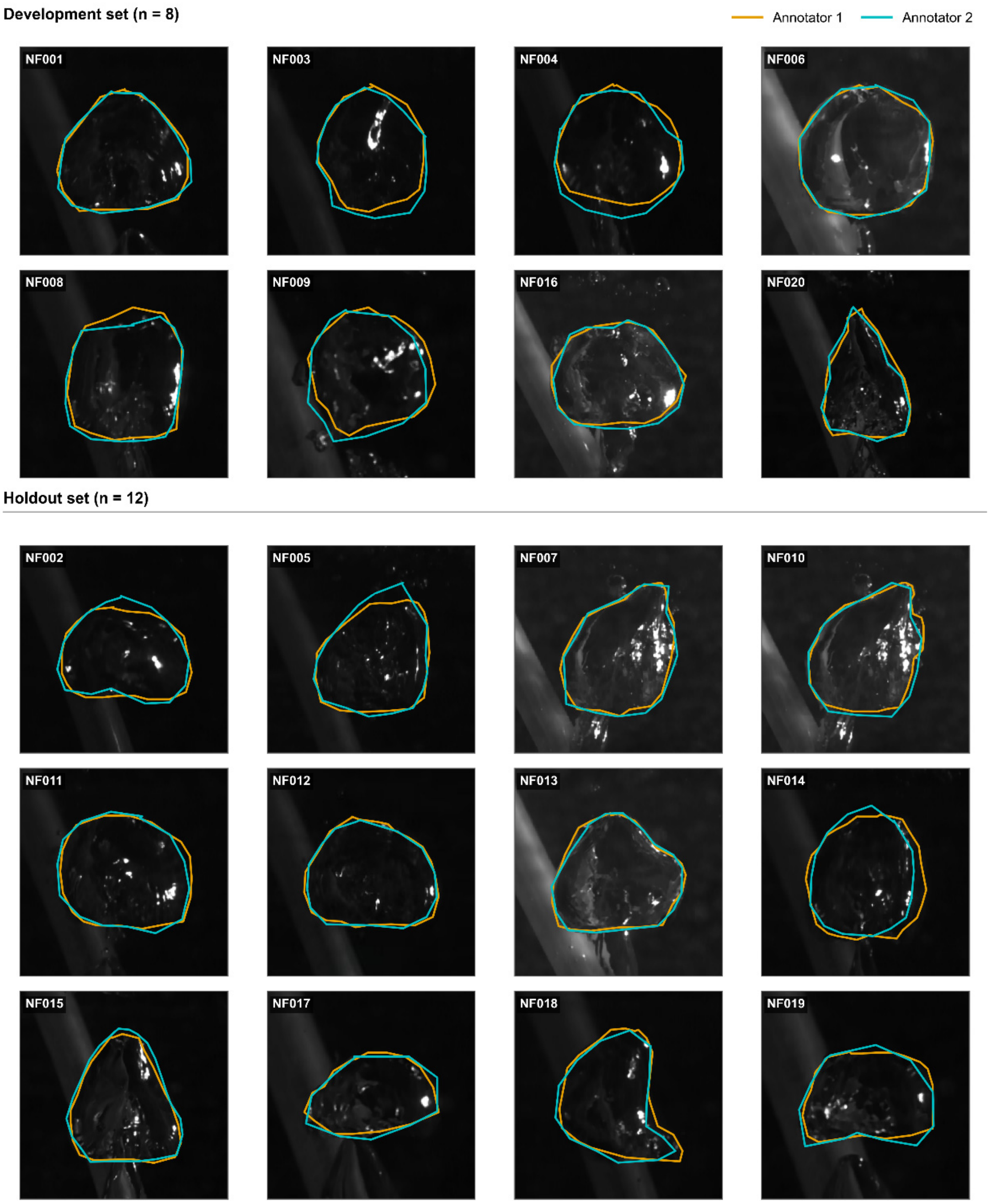

Figure S1. Independent manual annotations of the 20 near-field validation images. The outer contours delineated independently by Annotators 1 and 2 are shown in orange and cyan, respectively. Images are identified by blinded sample IDs and grouped into the development set (n = 8) and holdout set (n = 12). Square crops were applied consistently around the union of the two annotated contours to improve visibility; no local intensity adjustments were performed. The annotations were used to derive the fused image-based edge positions for the manual-reference comparison and do not constitute absolute physical ground truth.
