# Supplementary material for: Boundary-Transition Characterization in High-Speed Underwater Bubble Imaging Under Broadband and Narrow-Band Illumination
Source: Sensors (Basel). 2026 Aug 31;26(17):5546. doi: 10.3390/s26175546 (PMC13568190; doi:10.3390/s26175546)
Supplement: Supplementary file 1 [file sensors-26-05546-s001.zip › Supplementary File-Figure S2-round 2.pdf]

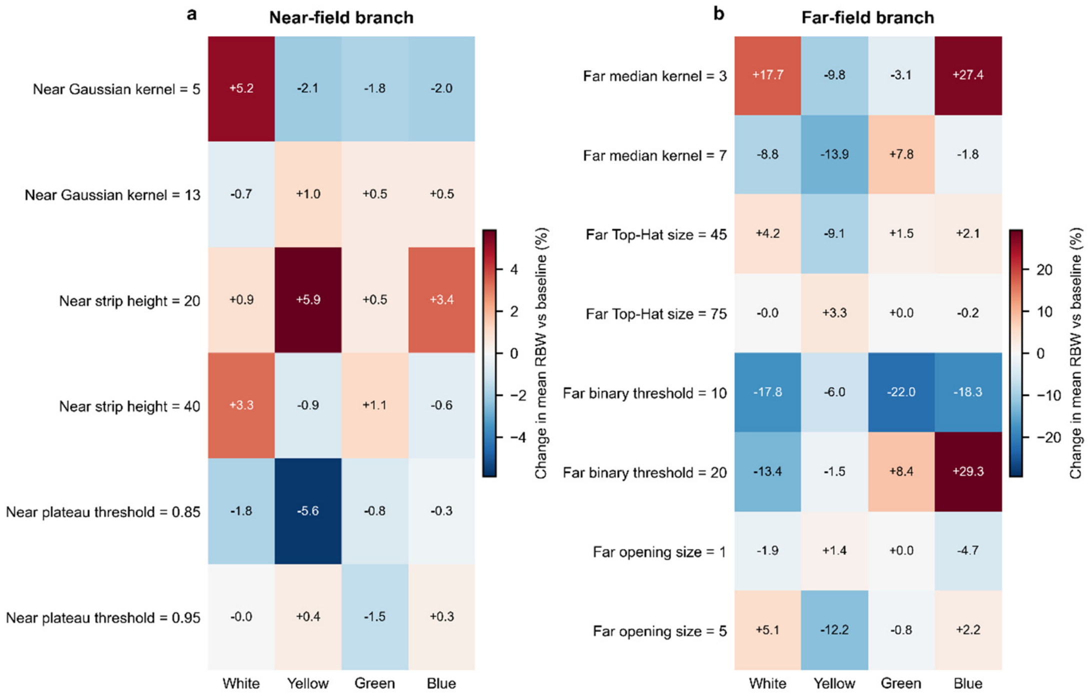

Cells show the percentage change in mean RBW relative to baseline; positive values indicate higher RBW.

Figure S2. One-factor-at-a-time sensitivity analysis of the RBW-processing parameters. (a) Near-field parameter perturbations. (b) Far-field parameter perturbations. Each cell shows the percentage change in mean RBW relative to the corresponding baseline condition; positive values indicate increased mean RBW. Each setting was evaluated using 20 frame-level subsamples per source condition, with one acquisition run available for each condition. All settings retained 100% valid prediction coverage. The figure summarizes parameter sensitivity within the available runs and does not provide between-run inference.
