# Supplementary material for: Boundary-Transition Characterization in High-Speed Underwater Bubble Imaging Under Broadband and Narrow-Band Illumination
Source: Sensors (Basel). 2026 Aug 31;26(17):5546. doi: 10.3390/s26175546 (PMC13568190; doi:10.3390/s26175546)
Supplement: Supplementary file 1 [file sensors-26-05546-s001.zip › Supplementary File-Table S1-round 2.pdf]

Supplementary Table S1. Multi-metric profile-level boundary-localization comparison on the 12-image near-field holdout set.

**Method**	**Mean BLE, px (95% bootstrap interval)**	**NBLE, %**	**P95 BLE, px**	**Precision@10**	**Recall@10**	**F1@10**	**Coverage, %**
Proposed P90 estimator	36.05 (27.58–43.71)	23.61	112.55	0.391	0.370	0.380	94.5
Canny	25.08 (18.17–32.42)	13.38	70.00	0.296	0.288	0.292	97.3
Otsu	39.76 (26.61–50.44)	22.32	118.70	0.188	0.178	0.183	94.5
Sobel	23.17 (17.74–27.54)	13.83	56.60	0.315	0.315	0.315	100.0
Prewitt	22.03 (16.46–26.53)	12.69	56.20	0.329	0.329	0.329	100.0
LoG	26.68 (19.89–32.44)	15.46	65.75	0.274	0.274	0.274	100.0
DexiNED	21.34 (15.72–26.69)	11.56	47.90	0.342	0.342	0.342	100.0

Note: BLE denotes the absolute profile-level boundary localization error, and NBLE denotes BLE normalized by the corresponding reference profile diameter. Precision@10, Recall@10, and F1@10 were calculated using a 10-pixel one-to-one matching tolerance. A valid prediction within the tolerance was classified as a true positive. A valid prediction outside the tolerance was classified as a false positive and left the corresponding reference unmatched. Prediction failures contributed to false negatives. Manually ineligible boundaries were excluded. Mean BLE and NBLE were calculated from valid predictions, while coverage denotes the proportion of the 73 eligible

observations for which a valid estimate was returned. The 95% bootstrap intervals summarize image-level resampling variability within the fixed 12-image holdout set and do not represent uncertainty across independent experimental runs.
